# Honey Bee *PTEN* – Description, Developmental Knockdown, and Tissue-Specific Expression of Splice-Variants Correlated with Alternative Social Phenotypes

**DOI:** 10.1371/journal.pone.0022195

**Published:** 2011-07-14

**Authors:** Navdeep S. Mutti, Ying Wang, Osman Kaftanoglu, Gro V. Amdam

**Affiliations:** 1 School of Life Sciences, Arizona State University, Tempe, Arizona, United States of America; 2 Department of Chemistry, Biotechnology and Food Science, University of Life Sciences, Aas, Norway; Martin-Luther-Universität Halle, Germany

## Abstract

**Background:**

Phosphatase and TENsin (PTEN) homolog is a negative regulator that takes part in IIS (insulin/insulin-like signaling) and Egfr (epidermal growth factor receptor) activation in *Drosophila melanogaster*. IIS and Egfr signaling events are also involved in the developmental process of queen and worker differentiation in honey bees (*Apis mellifera*). Here, we characterized the bee *PTEN* gene homologue for the first time and begin to explore its potential function during bee development and adult life.

**Results:**

Honey bee *PTEN* is alternatively spliced, resulting in three splice variants. Next, we show that the expression of *PTEN* can be down-regulated by RNA interference (RNAi) in the larval stage, when female caste fate is determined. Relative to controls, we observed that RNAi efficacy is dependent on the amount of *PTEN* dsRNA that is delivered to larvae. For larvae fed queen or worker diets containing a high amount of *PTEN* dsRNA, *PTEN* knockdown was significant at a whole-body level but lethal. A lower dosage did not result in a significant gene down-regulation. Finally, we compared same-aged adult workers with different behavior: nursing vs. foraging. We show that between nurses and foragers, *PTEN* isoforms were differentially expressed within brain, ovary and fat body tissues. All isoforms were expressed at higher levels in the brain and ovaries of the foragers. In fat body, isoform B was expressed at higher level in the nurse bees.

**Conclusion:**

Our results suggest that *PTEN* plays a central role during growth and development in queen- and worker-destined honey bees. In adult workers, moreover, tissue-specific patterns of *PTEN* isoform expression are correlated with differences in complex division of labor between same-aged individuals. Therefore, we propose that knowledge on the roles of IIS and Egfr activity in developmental and behavioral control may increase through studies of how *PTEN* functions can impact bee social phenotypes.

## Introduction

Insulin/insulin-like signaling (IIS) and epidermal growth factor receptor (Egfr) are important and highly conserved signal transduction pathways, spanning from yeast to fruit flies to humans [1],[2],[3],[4]. Research on invertebrate model organisms shows that many physiological processes are influenced by these pathways, including nutrient metabolism, growth, development, reproduction, and aging. Thus, IIS and Egfr cascades play major roles in invertebrate life-history regulation [1]. Also, in vertebrate systems, IIS and Egfr are part of the energy sensing systems of individual cells, and defects in these pathway components can lead to serious illness, including growth abnormalities, diabetes, and cancer [4],[5],[6].

The tumor suppressor gene *PTEN* is a dual specificity phosphatase that is conserved from nematode worms to humans [1],[7],[8]. PTEN can down-regulate both IIS and Egfr by dephosphorylating PI(3,4,5)-tris-phosphate to PI(4,5)P2, making it a direct antagonist of phosphoinositide-3-kinase (PI3K). Thereby, PTEN acts antagonistically to the growth-promoting signals from the activated insulin receptor [9],[10] and Egfr [3],[4]. The *D. melanogaster PTEN* homolog, *dPTEN* plays a critical role in regulation of cell proliferation, cell size, and organ/tissue size during development [11], and *dPTEN* homozygosity and trans-homozygosity causes lethality during embryonic and early larval stages [10]. In the nematode worm *Caenorhabditis elegans*, the *PTEN* homolog *daf-18* regulates dauer formation, i.e. developmental and life-extending arrest during the ‘L1’ larval stage [12]. Loss of *daf-18* bypasses this arrest and results in inappropriate growth [12]. *PTEN* has been studied primarily for its effects on longevity and cell size, in both worms and flies [10],[13],[14]. Yet, the recent insight that *PTEN* influences complex behavior, even in humans [15], suggests that research should be expanded to models of social behavior. The honey bee *A. mellifera* provides an attractive model for studies of molecular mechanisms that contribute to variation in social phenotype.

In honey bees, female larvae can develop into two reproductive castes: fecund queens or essentially sterile workers. During larval ontogeny, a bee goes through five larval instars, and female caste fate is determined in the 3^rd^ instar by nutrition. Adult workers that exhibit nursing behavior control the food provisions of the larvae. A queen-destined larva receives nutrient-rich diet (i.e. food rich in royal jelly) throughout development, whereas worker-destined larvae receive a less nutrient-rich diet from the 3^rd^ instar and onward. Larvae respond to this difference in nutrition by changing the expression of genes involved in IIS, Egfr and Target of Rapamycin (TOR) nutrient sensing cascades [2],[16],[17]. Consistent with nutrition being causal to female caste fate, queen-destined bees mostly up-regulate these genes compared to worker larvae [18],[19],[20], and larval gene-knockdown of the IIS associated *insulin receptor substrate* gene, *Egfr* gene and the central *TOR* gene causes queen-destined larvae to develop worker traits [17],[19],[21]. Wheeler and colleagues [18] reported that larval dietary manipulation affects *PTEN* transcript levels, but a function of PTEN in caste development was never tested directly.

Throughout adult life, worker bees perform tasks in a sequential, and thus age-associated, manner, resulting in a temporal division of labor. During the first weeks of adult life, workers typically stay inside the nest and take care of young larvae (nursing behavior). Thereafter, they go through a distinct behavioral shift to collect pollen, nectar or water in the field (foraging behavior) [22]. Although usually chronological in sequence, this behavioral ontogeny can be experimentally decoupled from age *per se*. If bees from a single age-cohort form a colony unit together (a single cohort colony), workers will divide labor so that some bees will nurse while others forage [23],[24],[25]. This model system is ideally suited to identify robust associations between gene expression, protein levels, endocrine physiology and complex behavior [26],[27],[28],[29],[30],[31],[32], and can be combined with dietary manipulations, pharmacology, and RNAi mediated gene knockdown to unravel causal relationships [33],[34],[35],[36],[37].

*PTEN* is alternatively spliced and has three and six isoforms in *D. melanogaster* [38] and the mosquito *Aedes aegypti*, respectively [39]. In mosquitoes, isoforms show developmental- and tissue-specific mRNA levels [39]. A putative *PTEN* ortholog was identified by the Honey Bee Genome Sequencing project and its transcript was detected in developing larvae [18]. Yet, the corresponding gene structure, as well as intra-organismal expression patterns, is unknown.

Here, we have cloned and characterized the *PTEN* gene. Alternate splicing was identified, resulting in three splice variants. We have used RNA interference (RNAi) to down-regulate *PTEN* expression in larvae, and show that the efficacy of the knockdown is dosage dependent and the phenotype associated with using the higher dosage of RNAi-inducing double-stranded RNA (dsRNA) is non-viable. Lower dosage of dsRNA did not lead to measurable *PTEN* down-regulation and produced a viable phenotype from larvae raised on queen diet. Finally, we have explored the tissue-specific gene expression patterns for the *PTEN* isoforms in adults, and show how these patterns correlated with social behavior using a controlled single cohort set up.

## Methods

### Bees

Wild type (unselected commercial stock) honey bees were used for all experiments. Bees were reared at the Honey Bee Research Facility on the ASU Polytechnic campus in Mesa, AZ.

### Cloning of *PTEN* isoforms

Total RNA isolated from worker larval instars (4^th^ and 5^th^) and from worker adult brain and fat body was used for cloning PTEN. Total RNA was treated with DNaseI (Ambion) and 5′ and 3′ RACE experiments were carried using the GeneRacer Kit (Ambion) according to the manufacturer's instructions. For the 5′ RACE following three reverse primers (5′ TCACAAATAGGTCGACTCCC 3′, 5′ TCCCCACGTGAGAGTAAACC 3′ and 5′ GTATCAAACGTGGCTGAACGTA 3′) were used in combination with the 5′ RACE supplied with the kit. For 3′ RACE following three forward primers (5′ TGATGTTGTCAAATTGTTGGAA 3′, 5′ TTTCCATGGAGGTCAAGGAT 3′ and 5′ CGCAAAGGAATGCATACGA 3′) were used in combination with the 3′RACE primers supplied with the kit. Four independent RACE experiments were carried out. RNA from different sources (larvae, adult brain and fat body) was used for RACE experiments. The PCR products were cloned into pCR® 4-TOPO® vector using TOPO TA cloning kit (Invitrogen), several clones (6–10 per experiment) were randomly picked and verified by sequencing. Subsequent to sequence analysis, full-length mRNA corresponding to the three *PTEN* isoforms was amplified and re-verified by sequencing.

### dsRNA synthesis and larval feeding

The dsRNA targeted all three isoforms and following forward and reverse primers were used for dsRNA synthesis 5′ TAATACGACTCACTATAGGGCGA TGATGTTGTCAAATTGTTGGAA 3′ and 5′ TAATACGACTCACTATAGGGCGA CGTATGCATTCCTTTGCGTA 3′ giving a product of 562 bp. T7 promoter sequence in underlined and dsRNA derived from GFP encoding sequence (503 bp) was used as a control [40],[41]. Queens from two wild-type background were caged for 24 h and three days later, newly emerged (12–18 h) old larvae (n = 100 per treatment group) were grafted into 24-well plates. First, the larvae were raised on a queen diet, the dsRNA was administered in the diet at either 150 µg/ml or 450 µg/ml concentration at 12 h intervals for two consecutive days. The details of the feeding regime for rearing queens were previously described by Patel and colleagues [19]. Due to methodological challenges, *in vitro* rearing typically does not yield a high proportion of queens. It is desirable that the frequency of successfully raised individuals with full queen morphology is higher than 50% before a diet is characterized as ‘queen-inducing’ diet. For the second set up, the feeding regime was modified and larvae were nutritionally restricted and fed every 24 h on the VS diet throughout larval ontogeny. This feeding regime yields primarily worker caste (Kaftanoglu O, Amdam GV, Page RE, unpublished data). The dsRNA was fed for four consecutive days at either 150 µg/ml or 450 µg/ml concentration at 24 h intervals**.** The larvae were collected 24 h after the final dsRNA feeding for knockdown verification and both set ups were independently replicated.

### Scoring morphological characters that distinguish queens, intercastes and workers

Queens were identified as having >100 ovarioles/ovary, notched mandibles, smooth stinger and absence of corbicula (pollen basket). Workers were identified as having 2–30 ovarioles, barbed stinger and presence of corbicula. Intercastes have characters reminiscent of queens but had smaller ovary size (ranging between 40–70 ovarioles/ovary). Ovariole scoring was carried out as described previously ([19] Detailed data on morphological characters are not shown, as their occurrence was in agreement with our earlier results reported in [19],[21].

### Preparing single cohort colonies

Two single cohort colonies were prepared in 4-frame standard Langstroth-size nucleus hives (19 inches in length and 19 1/8 inches in depth. (483 mm×232 mm). Queens (n = 2) were caged for 24 h to obtain newly laid eggs. The combs with the newly laid eggs were numbered and left in the colonies. These combs were removed 24 h prior to the emergence of adult workers and placed into an incubator at 35°C and 65% RH, in order to collect newly emerged bees. Single cohort colonies were established by placing about 7,000 newly emerged bees into the 4-frame nucleus hives. Each hive had 1 frame of honey, 1 frame of pollen, 2 fully drawn combs for queens to lay eggs.

### Sample collections

Foragers were marked in both single cohort colonies after 15 days. Foragers identified at the hive entrance when returning from foraging flights were marked on the abdomen with a dot of paint and then allowed to enter the hive and continue foraging. Five days later, nurses and marked foragers were sampled directly into liquid nitrogen. Nurses were identified on the brood with their heads inside the brood cells. For RNA isolation and mRNA quantification, materials from three bees were pooled to make up one biological sample per tissue (brain, ovaries, and fat body). Three such biological replicates were derived from each single cohort colony, to make up a total sample size of 6 for each tissue and behavioral group (nurse bee and forager).

### RNA Isolation and quantitative real-time RT-PCR (RT-qPCR)

For RNA was isolated from brain, ovaries and fat body using standard Trizol procedure except that the RNA was precipitated overnight in the presence of glycogen. For the RT-qPCR, total RNA was treated with DNaseI (Ambion) following standard instructions. RNA was diluted to 25 ng/µl and 2.0 µl was used a template. The RT-qPCR was run in triplicate (i.e. three technical replicates of the same sample on the same plate) using ABI Prism 7500 Applied Biosystems, and the data were analyzed using the comparative CT method [42] with *actin* (XM_623378) used as an reference gene. RT-qPCR conditions as described earlier by Wang and colleagues [43] were used. Following *PTEN* isoform-specific qPCR primers were used: PTEN_A Fp 5′ TCTGCATCTCTGGTGGTGAA 3′ and PTEN_A Rp 5′ TTGTGGTTTGCCGATGACTA 3′; for the B isoform, PTEN_B Fp 5′ ACCATGCATACAATAGGAAATGG 3′ and PTEN_B Rp 5′ ACAAATAGGTCGACTCCCCTGTGT 3′ and for the C isoform, PTEN_C Fp 5′ AAGCGGACAGCAGTGAATG 3′ and PTEN_C Rp 5′ AAAAATGTGTCCGCTGGTTT 3′. The amplification products were verified by sequencing prior to quantification. For *actin*, forward and reverse primers were Fp 5′ TGCCAACACTGTCCTTTCTG 3′ and Rp 5′ AGAATTGACCCACCAATCCA 3′ respectively. Negative control (without reverse transcriptase) for every sample was used to verify that the RT-qPCR assay was not confounded by DNA contamination or primer dimers. To determine the primer efficiencies, we checked melting curves for each set of primers and run the PCR products on agarose gels. Each primer pair had a single peak in melting curve analysis and a single sharp band of expected size on the agarose gel. Additionally, amplification curves of each *PTEN* isoform paralleled with those of *actin*, which indicated that primers for each gene had equal and comparable efficiencies.

### Statistics

For gene knockdown verification, *PTEN* expression levels were log transformed [43],[44] and Main Effect ANOVA was used for statistical analysis after validating that the data conformed the assumptions of Levene's test. Treatment group and qPCR plate (technical factor) were categorical predictors. Adult expression data were also log transformed and main effects ANOVA was conducted separately for each tissue. Behavioral caste, qPCR plate and colony were categorical predictors. Comparisons were not made between tissues or between isoforms because i) *actin* transcript levels (our reference gene within tissue) can be assumed to vary between tissues, and because ii) amplification efficacies may differ between the three isoform-specific primer sets. The data conformed to assumptions of ANOVA, as determined by Levene's tests. Fishers' LSD post hoc tests were used to identify the pattern of significance for each isoform. Statistica 6.0 (StatSoft) was used for all analyses. For ovary and adult wet weight, the data was not normally distributed, therefore we used the non-parametric Kruskal-Wallis test, and thereafter Mann-Whitney *U* tests for post hoc comparisons between the treatment groups.

## Results

### Identification of alternate splice variants

**Figure 1:**
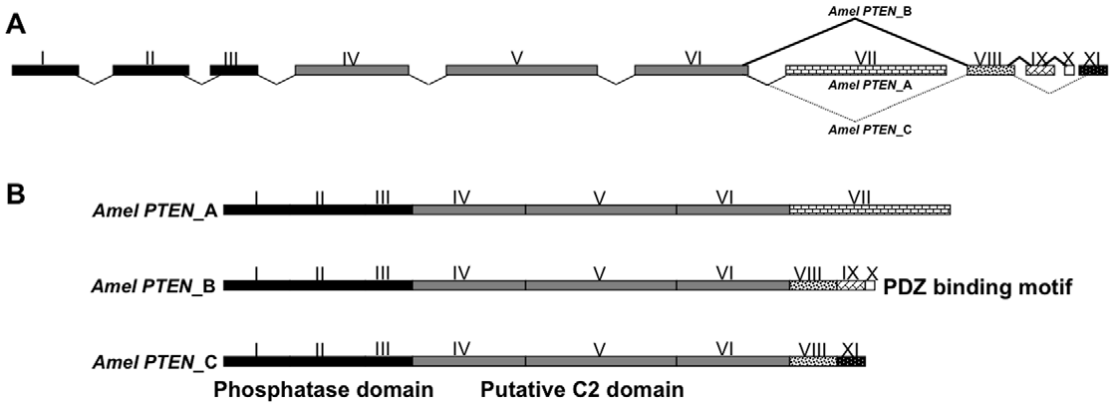
Schematic diagram of the organization of the honey bee *PTEN* gene. (A) cDNA sequences of three honey bee *PTEN* isoforms (A, B and C) were compared to the genomic sequences gleaned from the Honey Bee Genome Resources database (http://www.ncbi.nlm.nih.gov/genome/seq/BlastGen/BlastGen.cgi?taxid=7460) to define the exons (including alternative exons) and introns. (B) Three alternate splice forms were cloned and their structure is shown.

**Figure 2:**
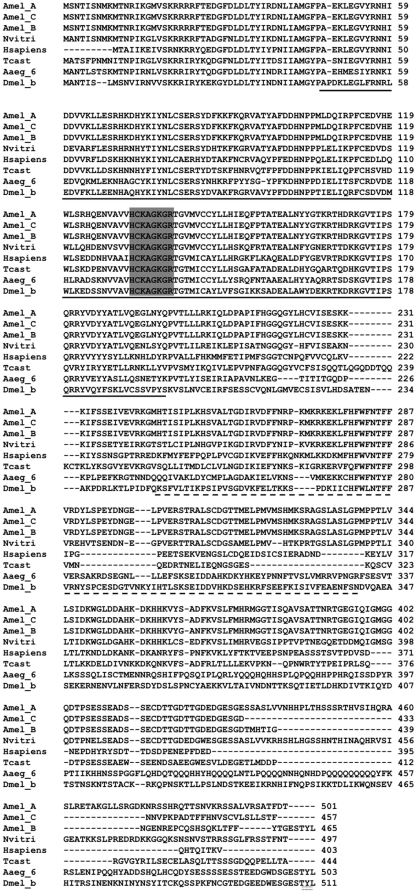
Alignment of the honey bee PTEN proteins toward other known PTEN amino acid sequences. The honey bee isoforms were aligned with the sequences of *D. melanogaster* (AF161258), *Nasonia vitripennis* (NP_001128398), *Tribolium castaneum* (XP_974994), *Aedes aegypti* (ABM21568) and *Homo sapiens* (NP_000305) using ClustalW version 1.82. The PTEN signature motif (HCXXGXXR) is highlighted (grey), the phosphatase domain underlined with a solid black line, the C2 domain underlined with a dashed line, and PDZ binding motif is double underlined. The Genbank accession numbers for three honey bee PTEN isoforms A, B and C are FJ_969918, FJ_969919 and FJ_969920, respectively.

A single honey bee *PTEN* gene was identified based on in silico genomic analysis by the Honey Bee Genome Consortium [45]. We used RACE (Rapid Amplification of cDNA Ends) to clone alternate splice variants of this gene, and to demonstrate that the gene, overall, contains eleven exons and ten introns. The intron/exon organization of *PTEN* is not shared between honey bee, *D. melanogaster,* mosquitoes (*A. aegypti and Anopheles gambiae*), *C. elegans* and human sequences ([39] and our Figs. 1 A and B). Despite these differences at the nucleotide level, the encoded proteins show relatively high amino acid sequence homology (Fig. 2). The degree of shared identity is 47% to *D. melanogaster*, 51% to *A. aegypti*, 37% to *C. elegans*, and 45% to the human proteins. The highest degree of identity is toward another hymenopteran insect, *Nasonia vitripennis* (73%).

As determined before for *D. melanogaster* [38], *A. aegypti* [39] and human [46], we found that the honey bee *PTEN* gene is alternatively spliced. Three splice variants were cloned (Fig. 1B). All isoforms encode the putative phosphatase domain (residues 52–198), essential for its activity as a tumor suppressor in humans [47] and the putative C2 lipid binding domain (residues 244–338), which has affinity to phospholipid membranes [47],[48]. The PTEN signature motif representing active site residues ‘HCXXGXXR’ (HCKAGKGR in honey bee) [48] was found at position 132–137 in all honey bee PTEN splice variants, and thus it is identical between all the available insect sequences so far (Fig. 2). The signature motif is also present in tyrosine phosphatases and dual specificity protein phosphatases but no sequence homology occurs outside this motif between these phosphatases and PTEN [49],[50]. The PTEN PDZ binding motif (XTXL/V), which has a potential role in protein-protein interaction [49],[50], was only present in honey bee PTEN isoform B. This finding is similar to the data on fly and mosquito PTEN, where only one isoform with PDZ binding motif was detected [38],[39]. Furthermore, of the three honey bee PTEN isoforms, only isoform A encodes multiple serine and threonine toward the C-termini that provide sites for post-transcriptional modification, particularly for phosphorylation (Fig. 2). Honey bee PTEN isoform C, thereby, was characterized as lacking both the PDZ binding motif (isoform B-specific) and the multiple serine and threonine residues (isoform A-specific).

### Effects of reducing *PTEN* gene expression during larval development

Knockout of *PTEN* in flies and mice is embryonic lethal [51],[52] and most of the studies to gain insights into PTEN function during development have being conducted on mutants of eye [10] or wing [51] tissue generated by somatic recombination. Here, we combined *in vitro* (laboratory) rearing of 1^st^–5^th^ instar honey bee larvae with suppression of *PTEN* activity by introduction of dsRNA against the *PTEN* gene in the larval food. The *PTEN* dsRNA targeted all the three isoforms of the gene (180–741 bp). Previously, we have used the *in vitro* dsRNA feeding technique to reduce larval expression of *TOR* and the *insulin receptor substrate, IRS* [19],[21]. Feeding of dsRNA is also successful in down-regulating gene expression in *C. elegans*, termites, tsetse fly and ticks [53],[54],[55],[56]. In our study, we tested two amounts of *PTEN* dsRNA in both of two diets to study efficacy and effects of *PTEN* gene knockdown. Each dsRNA/diet combination was replicated twice.

**Figure 3:**
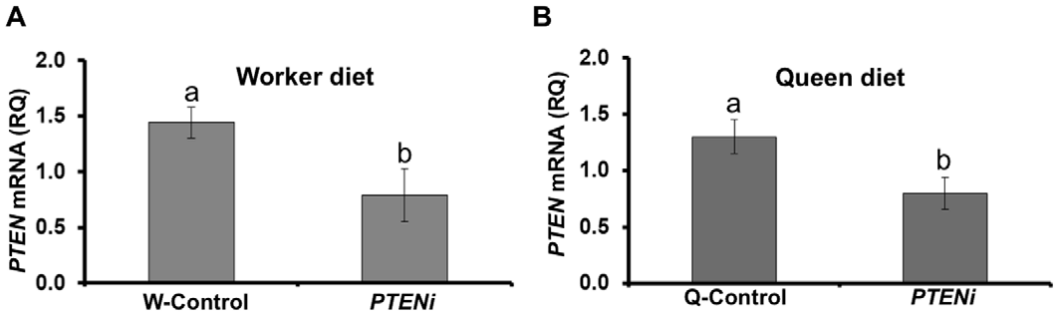
*PTEN* RNAi during larval development. Test of gene knockdown in honey bee larvae fed worker (A) vs. queen (B) diet in each of two separate experiments (n = 24). The larvae were fed with a higher dosage (450 µg/ml) of dsRNA which elicited significant *PTEN* knockdown (A,B). Bars represent mean ± s.e, different letters (a, b) denote significantly different groups, main effect ANOVA, p<0.05).

Larvae (n = 100, per treatment group) were either given a diet made primarily of royal jelly that supports queen development (queen diet) [19] or reared on a modified diet that supports worker development (worker diet) (see Methods for details). Additionally, we also tested the dosage dependent RNAi response using two different dsRNA dosages (150 µg/ml and 450 µg/ml). First, the diets contained dsRNA at a concentration of 450 µg/ml and were fed over two or four consecutive days depending upon the diet (details in the methods). Corresponding control groups were raised on both diets mixed with dsRNA against green fluorescent protein (GFP) sequence, which does not share homology with genes in the honey bee genome. This approach led to a significant reduction in *PTEN* expression in the animals reared on worker diet (main effects ANOVA: F _(1,21)_ = 5.55, p = 0.03, Fig. 3A) and on queen diet (main effects ANOVA: F _(1,43)_ = 5.40, p = 0.03, Fig. 3B). As before, the controls developed normally into either queens (>50%) or workers (>70%), depending upon the diet. Regardless of diet, however, none of the *PTEN* knockdowns completed metamorphosis. When they reached the pupal stage, *PTEN* RNAi phenotypes had body region deformities, such as distorted heads, thoraces and abdomens. Mortality was 100% within 5 days after the onset of pupation. Second, we tested a lower dose of dsRNA (150 µg/ml). This approach did not affect *PTEN* expression at the whole-body level irrespective the rearing conditions (Fig. S1A, B). As before, the controls developed normally into either queens or workers depending upon the feeding regime. Whereas, the larvae reared on queen diet and this low dosage emerged with intercastes characteristics (queen/worker intermediates) (See Fig. S2), while those reared on worker diet failed to complete development.

### Expression profiling of brain, ovary and fat body in different behavioral groups

**Figure 4:**
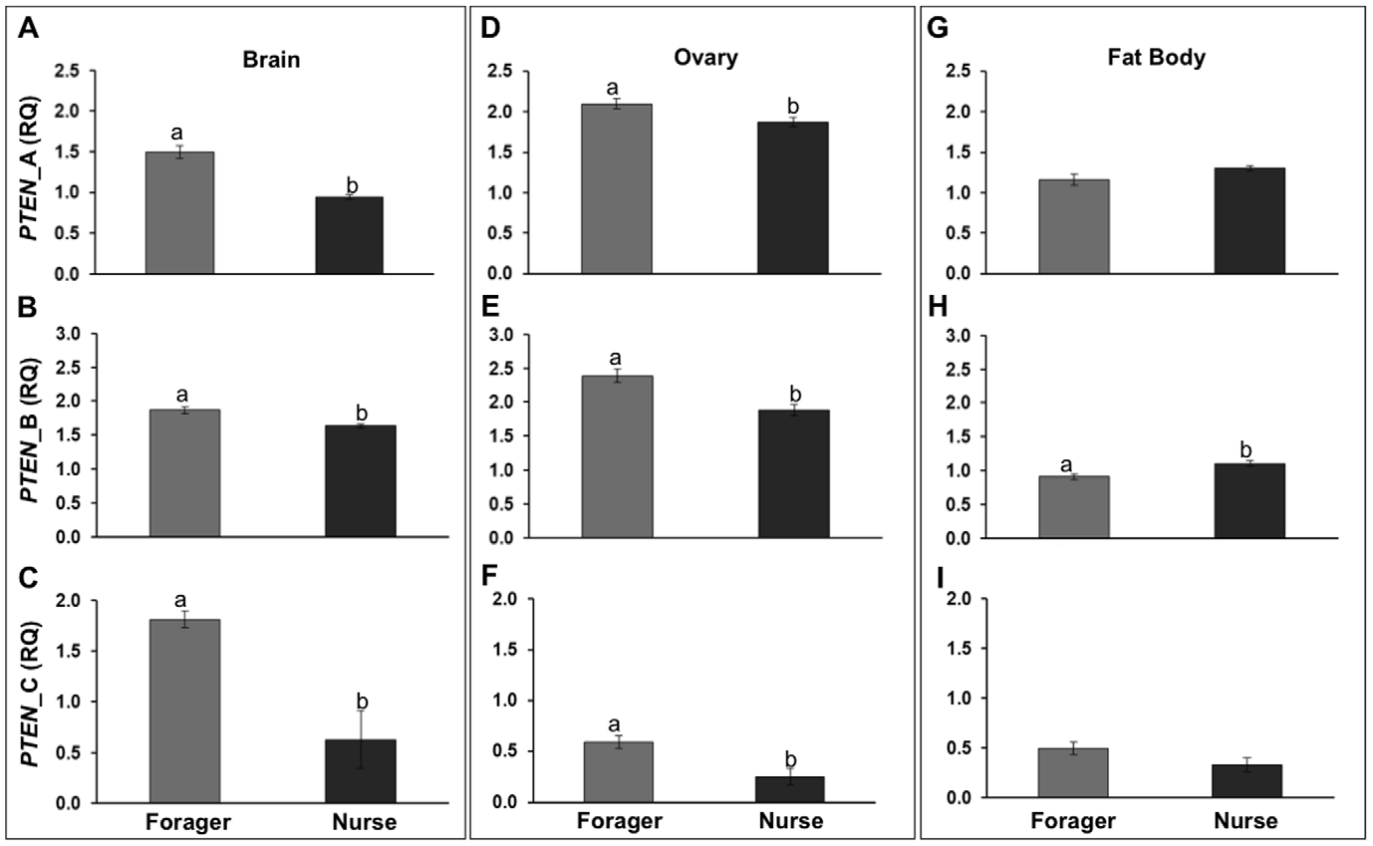
*PTEN* isoform- and tissue-specific expression in adult forager and nurse bees of the same chronological age. RT-qPCR was used to determine isoform-specific *PTEN* transcript levels in (A–C) Brain; (D–F) Ovary; and (G–I) Fat body. Age matched (20-day old bees) nurses and foragers from single-cohort colonies were used for the expression analysis. All RT-qPCR samples were run in triplicate. Brain, ovaries and fat body from 3 individual bees were pooled by tissue to make up one biological sample (n = 6). Bars represent mean ± s.e, different letters (a, b) indicate significant differences as determined with a Fisher LSD post-hoc test, p<0.05.

Using two replicate single cohort colonies, we studied *PTEN* mRNA expression in honey bee nurses and foragers (Fig. 4, Table 1). We found that all honey bee *PTEN* isoforms were expressed at a significantly higher level in the brains and ovaries of foragers compared to age-matched nurses (Fig. 4, A–F, see the legend for details on the statistics). In contrast, only *PTEN* isoform B was significantly elevated in the fat body of nurses when compared to age-matched foraging bees (Fig. 4H).

**Table 1:** Statistical analysis using main effects ANOVA.

Tissue	Test	Main effect	F value	d.f.	p
**Brain**	ANOVA	Behavior	13.8	3	<0.01
		qPCR plate	0.12	3	0.94
		Colony	0.32	6	0.91
	Post hoc comparison of isoform b/w nurses and foragers	Isoform A			<0.005
		Isoform B			<0.01
		Isoform C			0.01
**Ovary**	ANOVA	Behavior	6.75	3	0.03
		qPCR plate	0.26	3	0.85
		Colony	0.72	6	0.64
	Post hoc comparison of isoform b/w nurses and foragers	Isoform A			0.04
		Isoform B			<0.005
		Isoform C			<0.005
**Fat body**	ANOVA	Behavior	16.7	3	<0.01
		qPCR plate	0.6	3	0.63
		Colony	0.8	6	0.58
	Post hoc comparison of isoform b/w nurses and foragers	Isoform A			0.11
		Isoform B			0.01
		Isoform C			0.08

Behavioral caste (nurse bee and forager), qPCR plate and colony were categorical predictors. Post hoc comparison using Fisher's LSD were used to determine the effect of behavioral caste on each isoform (see Materials and Methods for details). Significant differences are highlighted in green.

**Figure 5:**
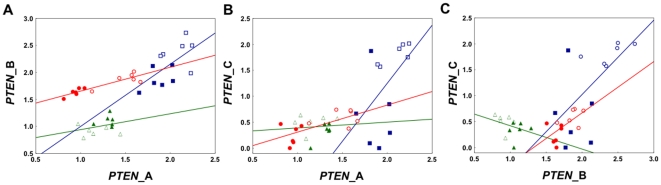
*PTEN* isoform-specific correlation plots by tissue. (A) Isoforms A and B, (B) Isoforms A and C (C) Isoforms B and C. Relative levels in brain (red circles), ovary (blue squares) and fat body (green triangles) are shown. Open circles, squares and triangles represent the forager data points while the closed symbols represent the nurse bee data points. As a general pattern, expression of *PTEN* isoforms is positively correlated in brain and ovary (Pearson's correlation, p<0.05), but not in the fat body of the workers (p>0.05).

Within tissue, we next plotted the correlative relationships between isoforms (Fig. 5). This approach allowed us to study putative associations between the transcript amounts, while taking into account that the absolute expression levels of the isoforms could not be directly compared. This is because we used a semi-quantitative measure for gene expression that is scaled separately for each gene product (see Methods). We found that *PTEN* isoforms A and B were significantly positively correlated in brain (Pearson's correlation, n = 6, p<0.0005) and in ovary (p<0.05), but not associated in fat body (p<0.4) (Fig. 5A). Similarly, isoforms A and C were positively correlated in brain (p<0.01) and ovary (p<0.01), but not in fat body (p<0.85) (Fig. 5B), and the pattern was repeated for isoforms B and C: significant correlation in brain (p<0.01) and ovary (p<0.007), but not in fat body (p<0.2) (Fig. 5C).

## Discussion

### *PTEN is* a conserved lipid and protein phosphatase

As a key negative affector of IIS and Egfr signaling, *PTEN* homologs have been identified in all eukaryote genomes sequenced so far. Our study has identified three *PTEN* isoforms in honey bees. Similar to fly and human *PTEN*, these isoforms all share the highly conserved PTEN signature motif ‘HC(X)_5_R’ [46],[48], the phosphatase domain [48] and the C2 domain [48]. Crystal structure provided insights into functional motifs responsible for mediating different PTEN functions [48]. Subsequent studies based on structure-functional analysis of these motifs, and, by mutating key residues within these motifs, strongly suggested that PTEN activity is required for normal growth and loss in its activity contributes to tumorigenesis [57]. These genetic approaches have also shown the link between the phosphatase domain and PI(3,4,5) P_3_ phosphatase activity [49],[58], and between the C2 lipid binding domain's phospho-lipid membrane affinity [47],[48] and Ca^2+^ dependent recruitment of kinases [59].

We found that the PDZ binding motif at the carboxy terminal is only present in honey bee PTEN isoform B. In mice, mutations in the PDZ domain greatly reduced PTEN ability to inhibit Akt/PKB signaling and lead to rapid degradation of the PTEN product, suggesting that PDZ domain stabilize PTEN through different protein-protein interactions [60],[61]. We also found that honey bee PTEN isoform A encodes putative phosphorylation sites toward the C-termini. Phosphorylation of these residues can result in conformation change that affects recruitment of PTEN-associated complexes to plasma membrane [62], and can further stabilize PTEN, control its subcellular location, and/or its association with other signaling molecules [49],[63],[64]. Thus, our data indicate how PTEN isoforms may have different stabilities that may be linked to different functions in honey bees.

### Knockdown of *PTEN* during larval development

We made a first effort to test honey bee PTEN function by down-regulating *PTEN* during larval development using RNAi. The dsRNA targeted all three isoforms during larval ontogeny. We used two different feeding protocols and two different dsRNA amounts. We fed the larvae with a high amount of dsRNA (450 µg/ml of each diet) (see Methods for details). This dosage produced a significant reduction of *PTEN* expression in larvae irrespective of the feeding regime (Fig. 3A, B). These knockdown larvae failed to complete development and died during pupation. Similar lethal phenotypes are also reported for the *PTEN* knockout mice [65] and flies [51]. We also tested a lower dsRNA concentration (150 µg/ml) (details in Methods). This approach did not reduce *PTEN* expression significantly (Fig. S1A, B). These results lead us to conclude that knockdown efficacy is dosage dependent. Dosage-dependent gene suppression is reported in mice, where efficacy of knockdown in different tissues (liver, kidney and lung) was directly proportional to dosage of siRNA administered [66]. Similar results are also reported for rat tissues [67] and in human cell lines [68].

Although *PTEN* expression was not reduced with lower dsRNA dosage, the phenotype was different from control: while larvae reared on worker diet failed to complete development, some larvae reared on queen diet completed metamorphosis and achieved intercaste or worker morphology. These adults had enlarged abdomens (see Fig. S2 B). Phenotypic effects in the absence of significant *PTEN* down-regulation might be explained by the target gene being affected in some regions and tissues but not in others. This heterogeneous response could make whole-body RNAi-detection difficult. In adult honey bees, where it is easier to conduct tissue specific studies of gene expression, regional RNAi efficacies are already confirmed [43]. Overall, our study did not provide evidence that PTEN is specifically involved in queen-worker differentiation because *PTEN* gene suppression was lethal in both phenotypes during development. Our inference from the results, therefore, is limited to PTEN playing a central role in nutrient sensing, presumably conferred by conserved effects on IIS and Egfr activity; and thus perturbation of *PTEN* can negatively impact larval development.

The significant down-regulation was achieved with a high dosage that resulted in non-viable phenotype. It can always be a concern that a non-viable phenotype is unspecific. However, many studies in honey bees and other organisms have highlighted the specificity of RNAi [54],[69],[70],[71],[72]. Recent results from *C. elegans*, moreover, suggest that regions must share 40 bp with 95% identity for off-target effects to occur [73]. A modified BLASTn search at the NCBI non-redundant database (with somewhat similar sequences option enabled) of our *PTEN* dsRNA region produced only one significant hit of 19 bp against another honey bee gene: a putative cytochrome P450. This makes off-target effects unlikely. In addition, controls reared on the same amount of *GFP* dsRNA developed normally, ruling out negative effects of dsRNA *per se*. Our study does not explicitly address the lethality of *PTEN* down-regulation, but the phenotype is well known from experiments in mice and flies [51],[52], where the effect is explained by various developmental defects reported in different tissues and organs [51],[52]. Future studies may explore if similar explanation can be found in bees. In this context, isoform-specific approaches may yield viable phenotypes and be more informative.

### *PTEN* expression and correlation to social behavior

We demonstrate that the honey bee *PTEN* isoforms are transcribed at different levels within the brain, ovaries and fat body of same-aged nurses and foragers (Fig. 4). In brain, all isoforms are expressed in higher levels in foragers. Since PTEN generally suppresses growth, our finding is not consistent with the volume increase of the neuropil of the mushroom body, a brain region that is generally expanded during honey bee foraging [74],[75]. This apparent discrepancy may be explained by the increase in neuropil volume being due to dendritic arborization rather than neurogenesis [76]. Dendritic arborization determines the nature and extent of innervation of a neuron in response to intrinsic and extrinsic signals and is a result of cytoskeleton changes in neurons [77]. PTEN is essential for proper localization of an F-actin-myosin II-based cytoskeleton in *Dictyostelium discoideum*, permitting the formation of filopodia necessary for both locomotion and chemotaxis [78]. In honey bees, increased locomotion and olfactory learning are both associated with foraging activities [79],[80]. Therefore, increased *PTEN* expression may be involved in cytoskeleton changes in mushroom bodies that occur during behavioral transition from in-hive work to foraging. However, we measured gene expression in the entire brain, and it is unclear how this overall transcript level reflects on dynamics in smaller sub-compartments like mushroom bodies.

Likewise, all isoforms show higher transcript levels in forager ovaries when compared to nurses of the same chronological age. In vertebrates, ovarian activity increases when *PTEN* expression is suppressed [81], and similarly, the propensity for ovary activation and egg-laying is increased in nurse bees compared to 21-day-old worker bees that are of forager age [82]. Thus, we hypothesize that elevated *PTEN* expression in the ovaries of forager bees contributes to their reduced propensity of reproductive activation [83].

In fat body (the abdominal adipose tissue), only the *PTEN* isoform B was transcribed at significantly different levels between the bees of different behavioral phenotype, and with increased expression in nurses. Nurse bee fat body also expresses vitellogenin (Vg) at high levels [25],[84],[85]. Vg is a yolk protein precursor and behavioral effector protein that is hypothesized to suppress IIS and perhaps Egfr signaling in honey bees [19],[86]. In general, IIS is anticipated to be reduced in nurse bee fat body [32]. Our result from *PTEN* isoform B is consistent with this expectation.

Finally, we found that, while the relative expression levels of all isoforms were correlated in brain and ovaries, none were correlated in fat body (Fig. 5). This pattern is largely driven by the expression dynamics described above: in brain and ovary, isoforms show highly variable expression levels that diverge consistently between nurse bees and foragers. In fat body, however, the variance is less pronounced and – with the exception of isoform B – the transcript levels do not diverge between the two behavioral groups.

### Honey bees as a model system to study *PTEN* function

**Figure 6:**
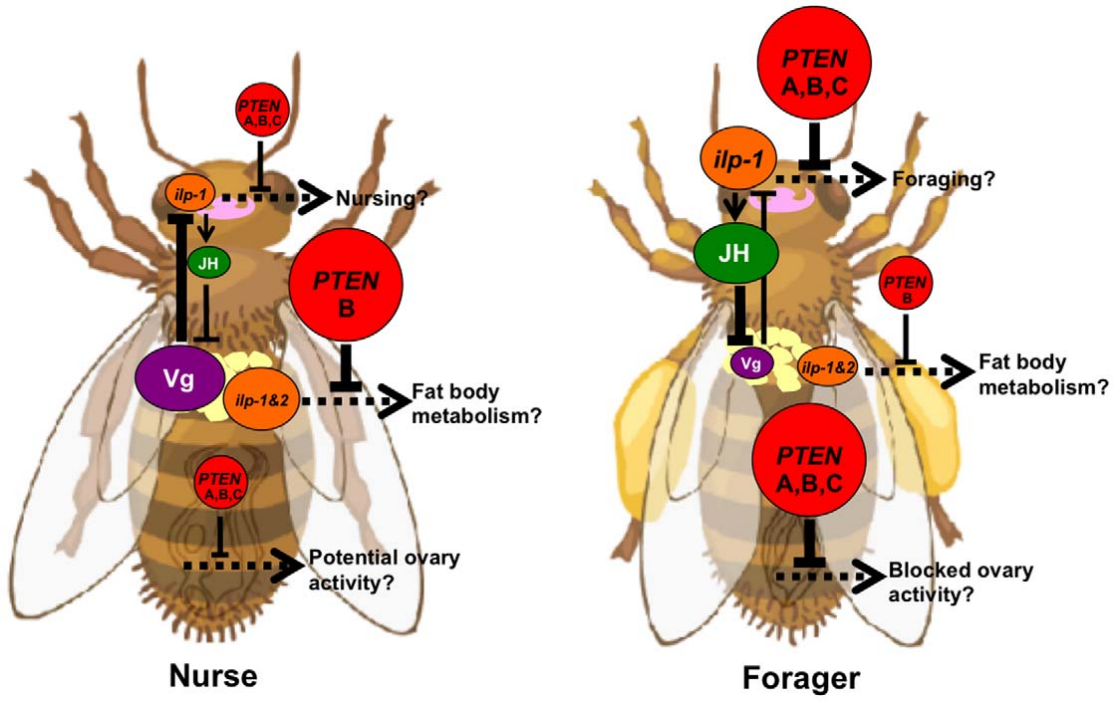
Possible tissue-specific *PTEN* functions in a regulatory network of honey bee behavior. In brain and ovary, *PTEN* (red circles) isoforms A, B and C, which could potentially down-regulate insulin/insulin-like signaling (IIS), are more abundant in foragers than in nurses. However, foraging behavior is positively associated with IIS via the release of *insulin-like peptide 1* (*ilp-1*, orange ellipse) in the brain (pink) [32]. *Ilp-1* may cause juvenile hormone (JH, green ellipse) levels to increase [91]; JH is also positively correlated with foraging behavior [94] and may enhance IIS by feedback suppression of vitellogenin (Vg, violet ellipse), a proposed negative regulator of IIS [34],[86],[92],[93]. These relationships contradict the repression of IIS by elevated *PTEN* in forager brain tissue. In contrast, suppressed IIS by *PTEN* in ovary tissue is consistent with the reduced reproductive propensity of foragers [31]. In fat body (yellow), *PTEN* isoform B, *ilp-1* and *insulin-like peptide 2* (*ilp-2*) are elevated in nurses compared to foragers (K. Ihle, unpublished data; and results in this paper), but effects on metabolic biology are currently unclear. The *ilp* gene products from fat body or brain may also take part in remote signaling to other organs [32]. In this illustration, larger-size circles/ellipses, and thicker arrows (positive)/blocked arrows (negative) denote higher levels of expression, enhancement and suppression, respectively. Dotted arrows indicate the yet unresolved effects on worker phenotypes.

Honey bees provide a model for understanding the molecular mechanisms that regulate complex behavior. In insects as well as mammalian systems, behavior can be affected by physiological feedback between brain, gonad and adipose tissue [26],[87],[88],[89],[90],[91]. This feedback is at least partly linked to nutrient- and energy sensing signals. In honey bees, IIS, partly through *insulin-like peptides* (*ilp*-*1* & *2*), can form a complex regulatory network influencing social behavior, which includes Vg and juvenile hormone (JH) (Fig. 6) [32],[86]. Reduced Vg can accelerate foraging behavior through feedback with JH, a systemic hormone with pleiotropic effects on metabolic biology and development [34],[86],[92],[93]. *TOR* can facilitate *vg* gene expression [19], while *IRS*, a central gene in IIS, can affect food-related behavior — presumably in interplay with Vg and JH [43]. Our gene expression results suggest that *PTEN* may be involved in adult social behavior (Fig. 6). Since PTEN influences Egfr signal transduction, Egfr could potentially also influence this regulatory loop to modulate the behavioral repertoire of worker bees. However, the role of Egfr in adult behavior is yet to be determined for honey beees.

*PTEN* knockdown mice are characterized by brain overgrowth and deficits in female social behavior [15]. Mutant alleles are also associated with human behavioral disease [15]. Thus, researchers have already established links between PTEN activity and complex behavior. To understand these relationships more fully, future studies on honey bees may provide insights that are less accessible in vertebrate systems or difficult to probe in flies (*D. melanogaster*), which lacks complex social behavior. In this context, our isoform- and tissue-specific data provide a platform for future experimental planning.

## Supporting Information

Figure S1 ***PTEN*** **RNAi during larval development.** Test of gene knockdown in honey bee larvae fed worker (A) vs. queen (B) diet in each of two separate experiments (n = 24). The larvae were fed with a lower dosage (150 µg/ml). Compared to the controls, the low dosage of dsRNA did not lead to measurable *PTEN* down-regulation at the whole-body level, neither for the queen diet, (main effects ANOVA: F _(1,43)_ = 0.50, p = 0.48, A) nor for the worker diet (main effects ANOVA: F _(1,21)_ = 0.38, p = 0.54, A). For the queen diet treatment, the controls primarily emerged as queens (56%) relative to 28% intercastes (individuals with mixed caste traits) and 14% workers, while those that received *PTEN* dsRNA emerged with intercaste phenotypes (44%) or with worker traits (52%). The phenotypic distributions of the bees, thereby, were different between the control and the *PTEN* dsRNA-containing queen diets (Chi-square test: χ^2^ = 83.1, df = 2, p<0.0001). Bars represent mean ± s.e.
(TIF)

Figure S2 **Effect of** ***PTEN*** **RNAi on physiological characters.** (A) Adult wet weight at emergence. Q-controls were heavier than the bees fed *PTEN* dsRNA and the W-controls (n = 20). Intercastes were characterized by enlarged abdomen (Fig. S1A and B), lower adult wet weight (controls: 187.6–232.7 mg vs. *PTEN* dsRNA fed group: 156.9–221.6 mg; Mann-Whitney *U* tests, p<0.001, n = 20 per group, (B) Ovary size. Q-controls had larger ovaries than the bees fed *PTEN* dsRNA and the W-controls (n = 10). Ovary size (controls: 120–165 ovarioles/ovary vs. *PTEN* dsRNA fed group: 38–70 ovarioles/ovary, Mann-Whitney *U* tests, p<0.001, n = 10 per group). For worker diet treatment, the controls primarily emerged as workers (74%, versus ∼22% with intercastes characteristics) while those that received *PTEN* dsRNA failed to complete development (Fig. S2 A and B; a Chi-square test on these character distributions was not performed due to the missing adult data on the *PTEN* dsRNA fed group). Bars represent mean ± s.e, different letters (a, b or c) denotes significantly different groups (A and B, Kruskal-Wallis test followed by post hoc Mann-Whitney *U* test, p<0.001).
(TIF)
