# Supplementary figures and images for: Honey Bee PTEN – Description, Developmental Knockdown, and Tissue-Specific Expression of Splice-Variants Correlated with Alternative Social Phenotypes
Source: PLoS One. 2011 Jul 14;6(7):e22195. doi: 10.1371/journal.pone.0022195 (PMC3136494; doi:10.1371/journal.pone.0022195)

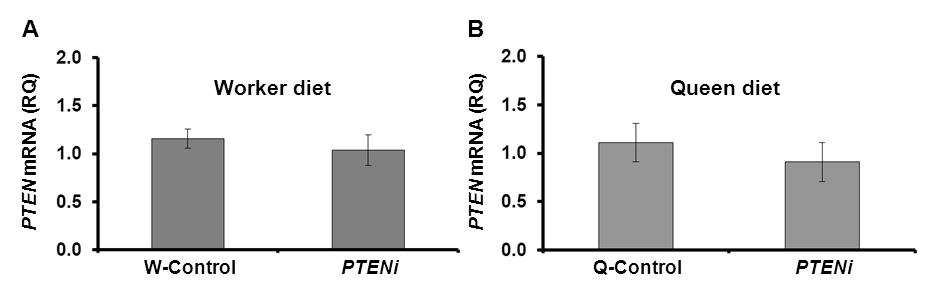

Supplement: Figure S1 — PTEN RNAi during larval development. Test of gene knockdown in honey bee larvae fed worker (A) vs. queen (B) diet in each of two separate experiments (n = 24). The larvae were fed with a lower dosage (150 µg/ml). Compared to the controls, the low dosage of dsRNA did not lead to measurable PTEN down-regulation at the whole-body level, neither for the queen diet, (main effects ANOVA: F (1,43) = 0.50, p = 0.48, A) nor for the worker diet (main effects ANOVA: F (1,21) = 0.38, p = 0.54, A). For the queen diet treatment, the controls primarily emerged as queens (56%) relative to 28% intercastes (individuals with mixed caste traits) and 14% workers, while those that received PTEN dsRNA emerged with intercaste phenotypes (44%) or with worker traits (52%). The phenotypic distributions of the bees, thereby, were different between the control and the PTEN dsRNA-containing queen diets (Chi-square test: χ2 = 83.1, df = 2, p<0.0001). Bars represent mean ± s.e. (TIF) [file pone.0022195.s001.tif]

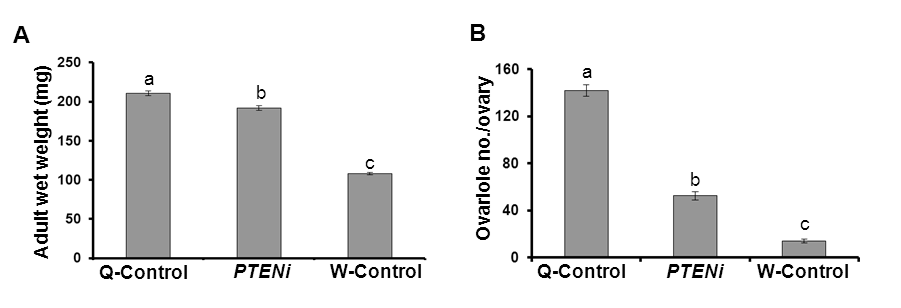

Supplement: Figure S2 — Effect of PTEN RNAi on physiological characters. (A) Adult wet weight at emergence. Q-controls were heavier than the bees fed PTEN dsRNA and the W-controls (n = 20). Intercastes were characterized by enlarged abdomen (Fig. S1A and B), lower adult wet weight (controls: 187.6–232.7 mg vs. PTEN dsRNA fed group: 156.9–221.6 mg; Mann-Whitney U tests, p<0.001, n = 20 per group, (B) Ovary size. Q-controls had larger ovaries than the bees fed PTEN dsRNA and the W-controls (n = 10). Ovary size (controls: 120–165 ovarioles/ovary vs. PTEN dsRNA fed group: 38–70 ovarioles/ovary, Mann-Whitney U tests, p<0.001, n = 10 per group). For worker diet treatment, the controls primarily emerged as workers (74%, versus ∼22% with intercastes characteristics) while those that received PTEN dsRNA failed to complete development (Fig. S2 A and B; a Chi-square test on these character distributions was not performed due to the missing adult data on the PTEN dsRNA fed group). Bars represent mean ± s.e, different letters (a, b or c) denotes significantly different groups (A and B, Kruskal-Wallis test followed by post hoc Mann-Whitney U test, p<0.001). (TIF) [file pone.0022195.s002.tif]
